# An emerging maestro of immune regulation: how DOT1L orchestrates the harmonies of the immune system

**DOI:** 10.3389/fimmu.2024.1385319

**Published:** 2024-06-19

**Authors:** Liam Kealy, Jessica Runting, Daniel Thiele, Sebastian Scheer

**Affiliations:** ^1^ Immunity Program, The Biomedicine Discovery Institute, Monash University, Clayton, VIC, Australia; ^2^ Department of Infection and Immunity, Luxembourg Institute of Health, Esch-sur-Alzette, Luxembourg

**Keywords:** DOT1L, immune system, epigenetic, immune regulation, methyltransferase

## Abstract

The immune system comprises a complex yet tightly regulated network of cells and molecules that play a critical role in protecting the body from infection and disease. The activity and development of each immune cell is regulated in a myriad of ways including through the cytokine milieu, the availability of key receptors, via tailored intracellular signalling cascades, dedicated transcription factors and even by directly modulating gene accessibility and expression; the latter is more commonly known as epigenetic regulation. In recent years, epigenetic regulators have begun to emerge as key players involved in modulating the immune system. Among these, the lysine methyltransferase DOT1L has gained significant attention for its involvement in orchestrating immune cell formation and function. In this review we provide an overview of the role of DOT1L across the immune system and the implications of this role on health and disease. We begin by elucidating the general mechanisms of DOT1L-mediated histone methylation and its impact on gene expression within immune cells. Subsequently, we provide a detailed and comprehensive overview of recent studies that identify DOT1L as a crucial regulator of immune cell development, differentiation, and activation. Next, we discuss the potential mechanisms of DOT1L-mediated regulation of immune cell function and shed light on how DOT1L might be contributing to immune cell homeostasis and dysfunction. We then provide food for thought by highlighting some of the current obstacles and technical limitations precluding a more in-depth elucidation of DOT1L’s role. Finally, we explore the potential therapeutic implications of targeting DOT1L in the context of immune-related diseases and discuss ongoing research efforts to this end. Overall, this review consolidates the current paradigm regarding DOT1L’s role across the immune network and emphasises its critical role in governing the healthy immune system and its potential as a novel therapeutic target for immune-related diseases. A deeper understanding of DOT1L’s immunomodulatory functions could pave the way for innovative therapeutic approaches which fine-tune the immune response to enhance or restore human health.

## Introduction

The methyltransferase (MT) Disruptor of telomeric silencing 1 (DOT1) was initially identified in budding yeast (due to its role in the maintenance of telomeric regions), which led to the discovery of its mammalian homolog DOT1-like (DOT1L). DOT1L is a 1537 amino acid protein, and in contrast to other known histone methyltransferases (HMTs), is the only known HMT that lacks a Su(var)3-9, Enhancer-of-zeste and Trithorax (SET) domain harbouring methyltransferase activity (1–3). Instead, DOT1L possesses an Adomet-binding motif, which catalyses the transfer of an S-methyl group from the cosubstrate S-Adenosyl methionine (SAM) to carry out the mono-, di- or trimethylation of lysine 79 within the globular domain of histone 3 (H3K79) (1–3). Since it specifically methylates a lysine – and not other possible amino acids, such as arginines – DOT1L is also known as a lysine methyltransferase (KMT) 4 (1, 4–6). DOT1L is the primary KMT responsible for H3K79 methylation, with its deletion resulting in the complete loss of H3K79 methylation (3, 7, 8). Most studies use the presence of H3K79me2 as a surrogate for DOT1L activity, as this antibody is widely accepted as more reliable and sensitive than any of the antibodies generated against DOT1L itself. In addition, it was shown in yeast that DOT1 cannot generate different states independently from each other, possibly suggesting functionally redundant roles for each state (7). With the aid of its cofactor ALL1-Fused gene from chromosome 10 (AF10), DOT1L is recruited to chromatin to carry out the methylation of H3K79 (9). Unlike other KMTs, DOT1L can methylate H3K79 in intact chromatin but not as free histone or histone peptides (10). Lysine demethylase (KDM)2B has been identified as a demethylase of H3K79me, however little is known about this in a primary cell context (11, 12). Other studies suggest that H3K79 methylation is merely lost through histone dilution via replication-dependent and independent manners, as well as nucleosome turnover (13).

In general, DOT1L is important for embryonic development, the DNA damage response, meiotic checkpoint control, mammalian erythropoiesis and cardiogenesis (6, 8, 14–20). Germline deletion of DOT1L is embryonically lethal between E9.5 and E10.5, resulting in defects in the yolk sack and heart (8). The discovery of DOT1L’s contribution to cancer progression, particularly in the context of MLL-rearranged leukaemia, was monumental to the field (21–23). It quickly became clear that the aberrant recruitment and targeting of DOT1L and the resultant skewing of H3K79 methylation patterns across the epigenome were the major drivers of oncogenesis in this type of cancer (24, 25). It was further established, via a suite of laboratory testing, that DOT1L inhibition resulted in dysregulation of the cell cycle and cell death in MLL-fusion-driven leukemic cells as well as resensitisation to chemotherapy in other types of cancer (19, 25–27). These breakthroughs launched a fleet of clinical trials designed to test the safety and efficacy of the DOT1L inhibitor Pinometostat (EPZ-5676) in patients with MLL-rearranged leukaemia (28, 29). Whilst these trials have shown promise for DOT1L inhibition as a cancer therapeutic, they have also highlighted the current lack of understanding regarding the contribution of DOT1L to normal homeostatic biology, such as within the immune system.

Investigation into the role of DOT1L in the context of the immune response has only recently come to light (**Table 1**). Our groups and others have provided the first evidence of a role for DOT1L in regulating adaptive immune responses (31, 34, 35, 42, 43). These studies collectively highlighted a role for DOT1L in B and T cell development, as well as lineage integrity and function. Other groups have also begun to characterise DOT1L in innate immune cell development and function including macrophages, bone marrow derived dendritic cells (BMDCs) and myeloid derived suppressor cells (MDSCs) (38–41). This review will aim to consolidate our current knowledge about DOT1L in the context of the immune system (summarised in **Table 1**), whilst also discussing known and potential hypothetical mechanisms by which DOT1L may be regulating these biological pathways. We further highlight the current technical limitations faced by researchers attempting to map the contribution of this key KMT across the immune network and propose future directions for research and therapeutic avenues involving the targeting of this unique immune orchestrator.

**Table 1:** The role of DOT1L in the immune system.

Cell type/subset		Method	Model	Role of DOT1L	Consequence	Reference
Lymphocytes	all	cKO	CreER-DOT1L^fl/fl^	Maintenance of hematopoiesis	Failure of functional stem cells and loss of hematopoietic cells	(30)
	all	cKO	VavCre-DOT1L^fl/fl^	Required for optimal lymphopoiesis	Moderate neutropenic and lymphopenic	(25)
CD4^+^ T cells	Th1	Inh	Ex vivo with small molecule inhibitor (SGC0946)	Limits Th1 program	Increased expression of IFN-γ and TNF	(31)
	Th2	cKO	Ex vivo with small molecule inhibitor (SGC0946)	Limits Th2 program	Increased IL-4/IL-13	(31)
	Th17	Inh	Ex vivo with small molecule inhibitor (SGC0946)	Limits Th17 program	Increased IL-17	(31)
	All T cells	Inh	Ex vivo with small molecule inhibitor (EPZ004777); tumour	Required for AMPK expression	Impaired AMPK expression, increased PD-1 expression	(32)
	All T cells	cKO	*In vivo*; CD4Cre-DOT1L^fl/fl^; inflammation, allergy, worm infection	Limits Th1 program	Increased expression of IFN-γ and TNF; decreased Th2 immune response	(33)
CD8^+^ T cells	all	Inh, cKO	Ex vivo small molecule inhibitor (EPZ004777); *In vivo*; CD4Cre-DOT1L^fl/fl^; tumour	Required for STAT5 expression	Decreased STAT5 expression and impaired T cell immunity	(34)
	all	cKO	*In vivo*; LckCre-DOT1L^fl/fl^	Prevents antigen-independent differentiation	Increased memory phenotype	(35)
	all	Inh, Trans	Ex vivo; DOT1L small molecule inhibitor (SGC0946); transfer *in vivo*; GVHD	Alleviates allogenic T cell responses	Decreased miR-181a leading to increased DUSP6 expression	(36)
NK cells	all	cKO	*In vivo*; Ncr1Cre-DOT1L^fl/fl^; tumour	Maintains lineage integrity	Increased intILC1 phenotype and decreased tumour control	(37)
Macrophages	all	RNAi, siRNA, Inh	Ex vivo; DOT1L silencing; small molecule inhibitors (EPZ5676)	Promotes IL-6 and IFN-β	Impaired IL-6 and IFN-β production following treatment with innate stimuli	(38)
	all	Inh, cKO	*In vivo*; LysMCre-DOT1L^fl/fl^ Ex vivo with small molecule inhibitors (SGC0946)	Controls lipid biosynthesis	Increase in hyper- inflammatory macrophages	(39)
DCs	BMDC	Inh	Ex vivo with small molecule inhibitors (EPZ004777)	Promotes FOXM1 expression	Increases BMDC maturation	(40)
MDSCs	all	Inh, Inj	*In vivo* and ex vivo with small molecule inhibitors (EPZ5676)	Promotes SOCS1 (inhibitor of STAT1 signalling pathway) expression	Enhances the suppressive function of MDSCs	(41)
B cells	all	cKO	*In vivo*; Mb1Cre-DOT1L^fl/f^, CD23-DOT1L^fl/fl^ Ex vivo with small molecule inhibitors (SGC0946)	Promotes B-cell development and GC formation	Reduced class switching; loss of GC B cells and reduced plasma cells. Decreased immunisation efficiency.	(42)
	all	cKO, Inh	*In vivo*; Mb1Cre-DOT1L^fl/f^, Ex vivo with small molecule inhibitors (EPZ5676)	Promotes B-cell development and GC formation	Reduced class switching; loss of GC B cells and reduced plasma cells. Decreased immunisation efficiency.	(43)
	B cells and T cells	H3K79me coverage	Ex vivo; ChIP for H3K79me2 in regions of rearrangement	Associated with light chain rearrangement	Descriptive: H3K79me2 is present at rearranging sites	(44)
	B cell line	H3K79me coverage, Inh, CRISPR/ Cas9	Cell line, EPZ004777, EPZ5676, gRNA	Cooperates with AID to achieve somatic recombination	Reduced SHM	(45)

mKO, conditional murine knockout; Inh, inhibitor (showing methyl-transferase dependency); Trans, transfer of inhibitor-treated cells in vivo; Inj, Injection of inhibitor into mice.

## General mechanisms

The most widely accepted molecular function of DOT1L is its methylation of H3K79, which has been associated with lineage-specific gene expression and is enriched at promoter-proximal regions of active genes (46–52). DOT1L inhibition reduces the expression of ordinarily highly transcribed genes and increases the expression of typically repressed genes (53). Contrarily, DOT1L-dependent H3K79me2 and H3K79me3 have also been correlated to silenced genes (54). Therefore, DOT1L could be associated with repressing and activating certain genes within the same cell (13, 20, 54–56). One study highlighted that a distinct subset of enhancers depends on H3K79 methylation for their promoter interactions (57). Further, H3K79 methylation could also be influenced by other epigenetic modifications, such as the ubiquitination of H2BK120 and histone deacetylation by HDAC1 (10, 58–61). Interestingly, cryo-electron microscopy of DOT1L showed that it can interact with H2BK120 ubiquitination in order to switch to a more active state (61). These findings reveal that DOT1L activity can be dynamically modulated by the local epigenetic landscape in an allosteric fashion. Cross-talk between H3K79me and other histone marks also appears to exist as *Jones et al.* observed a significant decrease in H3K9me2 and H4K20me3 following the loss of H3K79me (8). The implications and context-specific consequences of this cross-talk however requires further elucidation.

Recently, the scaffold protein Menin has been identified as a *bona fide* reader of DOT1L-dependent H3K79me2 (62). Menin was implicated in transcriptional regulation, as it can interact with numerous chromatin regulatory proteins (such as MLL1) and transcription factors (TFs) (63–66). Therefore, Menin may recruit other cofactors to H3K79me2-enriched chromatin (such as at intragenic enhancers) for gene regulation (62). Further characterisations of DOT1L-dependent H3K79 methylation in the context of other histone modifications (such as H3K27me3, H3K9me2 and H3K4me3, and histone deacetylation), and their connection with Menin, will offer further insight into both its function and dynamics alongside these other transcriptionally modulating marks.

Many methyltransferase-independent roles for DOT1L have also been reported. These include transcriptional initiation, nucleosome remodelling, histone exchange and H2B ubiquitination (67–71). In addition, DOT1L is implicated in the DNA damage response pathway by promoting the repair of double-strand breaks (8, 19, 72). In the absence of DOT1L mediated H3K79me, lung cancer cells are unable to repair DNA damage caused by division leading to accumulation of phosphorylation of ser 139 on histone H2AX (γH2AX) resulting in cell cycle arrest; a feature of cellular senescence (73). Contrary to these observations, DOT1L was also shown to facilitate the senescence-associated secretory phenotype (SASP) through H3K79me within the *Il1a* gene locus (74).

53BP1, a crucial component of double-strand DNA damage repair, requires DOT1L mediated H3K79 methylation for recruitment to damaged DNA (72). In the absence of DOT1L-mediated H3K79me, DNA is unable to be repaired, leading to decreased 53BP1 foci (72, 75, 76). Similarly, γH2AX is needed at the site of DNA damage and colocalizes with 53BP1 (72). Contrary to previous observations in lung cancer cells (76), γH2AX requires DOT1L-mediated H3K79me and its absence leads to decreased γH2AX levels and reduced DNA damage repair in models of induced DNA damage (76, 77). The contradiction between observations is largely due to the different models tested: whilst one is measuring the ability of a cell to repair damage in response to cell division where accumulation of γH2AX is unfavourable (76), the other is measuring repair in response to a mutagen where accumulation is favourable. In line with this protective role in facilitating DNA damage repair, *Dot1l* is often found to be highly mutated in human melanoma samples leading to inefficient repair of UV-induced DNA damage (77). Mechanically, DOT1L-mediated H3K79me is required to recruit the xeroderma pigmentosum complementation group C (XPC) complex for nucleotide excision repair (NER) (77, 78). Given DOT1L is often found to be increased in response to inflammatory cues, such as innate stimuli (38) and its defined role in DNA damage repair, it would be interesting to understand whether this is a result of increased cell death due to unrepaired dsDNA breaks.

Along with its role in regulating DNA damage repair mechanisms, it also appears that DOT1L has a role in maintaining genomic stability from observations made by *Barry et al.* in mouse embryonic stem cells (ESCs) (79). Following the deletion of DOT1L, ESCs can proliferate, however, upon differentiation these cells lose this ability to proliferate and are unable to die via apoptosis or undergo substantial changes in gene expression. Similar observations were observed by *Jones and colleagues* wherein deletion of DOT1L in ESCs led to decreased proliferation, telomere elongation and aneuploidy (8). Taken together, it is clear that DOT1L mediated H3K79me plays a diverse role in regulating cell cycle progression with substantial evidence suggesting DOT1L is needed for cell cycle progression (80).

Using a DOT1L methyltransferase mutant mouse model (*Dot1l*-MM), *Malcolm et al.* showed that early mammalian embryonic erythropoiesis is independent of DOT1L methyltransferase activity (81). Similar to systemic deletion of DOT1L in mice, *Dot1l*-MM were also embryonically lethal (E13.5), highlighting that DOT1L dependent H3K79me is still required during development. In a follow-up report, the same group reported that the primary function of DOT1L in hematopoietic progenitor cell development is to act as gene repressor, a function that can be independent from its methyltransferase activity (82). Both reports clearly suggest that DOT1L has methyltransferase-independent functions, which also contribute to embryonic development. It would therefore be highly interesting to assess the methyltransferase-independent functions of DOT1L in mature immune cells, wherein we might envision a role in scaffolding, linking, or limiting protein complexes and thereby regulating gene expression.

Given its broad role in DNA damage repair, cell cycle regulation, telomeric silencing and initiating transcriptional elongation (described earlier), it is unsurprising that DOT1L’s function is not limited to its enzymatic activity. DOT1L is capable of binding with other factors to form complexes known as DOT1L-containing Complexes (or DotComs) (83, 84). Known components of these DotComs include SIRT1 (85), AF9, ENL, AF10 (86), AF17, BCOR (84, 87) and the Wnt signalling components TRRAP, Skp1 and beta-catenin (83, 88). DotComs are thought to facilitate gene transcription via direct recruitment of RNA polymerase II or the repression of repressive complexes containing HDACs and SUV39H1 (85, 87). DotComs have been extensively studied as therapeutic targets in the context of MLL; where DOT1L activity is augmented to facilitate leukaemia gene expression (84), a role for DotComs in immune cell regulation has not been explored. Given that SIRT1 (89), SUV39H1 (90, 91), Wnt signalling (92) and BCOR (93) all play a role in immune cell regulation, it is likely that DotComs play a significant role in immune cell function or development and should therefore be the subject of future research.

Overall, these observations highlight a diverse role for DOT1L mediated H3K79me in regulating both sides of the same cellular process highlighting the need for further investigation. It is also possible that DOT1L is involved in recruiting certain TFs or chromatin regulators to particular loci in a lineage-specific manner; however, more evidence is required to substantiate these assumptions.

## Innate immune system

Macrophage polarisation states (M1 and M2) are unstable *in vivo*, where the extracellular milieu can change from pro- to anti-inflammatory. Subsequently, this milieu can change the epigenetic status of a cell, thereby defining the transcriptional profile and - in the end - changing the polarisation of macrophages (94). In particular, this can be observed in the tumour microenvironment (TME), where proinflammatory M1 and M2 macrophages are largely influenced by their location (95). It would therefore be of great interest to identify a component of the epigenetic family that could be inhibited to switch macrophages from M2 to M1 in the TME, subsequently providing a tool to overcoming a barrier for efficient immunotherapy. While it was not shown that DOT1L directly influences macrophage polarisation, DOT1L was shown to promote the production of IL-6 and interferon (IFN)-β but not of TNF-α in primary peritoneal macrophages and the cell line THP-1 upon triggering with TLR ligands (38). Contrary to these findings, Willemsen and colleagues reported that DOT1L limits macrophage hyperactivation and regulates lipid biosynthesis gene programs (39). The authors show that inhibition of DOT1L with the small molecule inhibitor SGC0946 and using bone marrow derived macrophages (BMDM) results in increased production of IFN-β in response to lipopolysaccharides (LPS), which is usually mediated by the DOT1L-dependent suppression of the SREBP pathway (39). It is possible that these contrary findings are a result of using different macrophage populations and it would therefore be interesting to assess this phenomenon in more detail in future experiments. The study by Chen and colleagues is also the only report that uses an antibody against DOT1L for ChIP-seq (38). However, ChIP-seq using an antibody against DOT1L in primary cells was not successfully reported elsewhere (including our laboratories) and may require additional validation and improvement of the antibody target specificity.

For CD8^+^ T cells, it was shown that DOT1L activity in the TME can be influenced by methionine availability as DOT1L’s affinity to methionine is low (34). As CD4^+^ T cells show an increased Th1 phenotype in the absence or after inhibition of DOT1L (31, 33), efforts should be directed to see whether depletion of DOT1L or the limited availability of methionine also has a direct influence on the polarisation of T cells and macrophages in the TME. Together, this hints towards the importance of DOT1L in maintaining macrophage function in the TME and possibly limiting excessive macrophage-mediated inflammation elsewhere. 

In a murine model of (*P. acnes*)-primed LPS224-induced fulminant hepatitis, *in vivo* inhibition of DOT1L using EPZ-5676 protected the mice from succumbing to infection. The authors observed a dependency on the presence of myeloid-derived suppressor cells (MDSC), as demonstrated through the depletion of these cells by injecting anti-GR-1 antibodies. Mechanistically, it was shown that the suppressive function of MDSCs was dramatically increased leading to the suppression of pathogenic CD4^+^ T cells, which contribute to disease progression (41). Interestingly, in this setting, inhibition of DOT1L with small molecule inhibitors did not have a direct effect on CD4^+^ T cells but only indirectly through MDSCs. However, this may be due to spatial or temporal restrictions during these experiments, as we previously showed that inhibiting DOT1L has a profound impact on CD4^+^ T cells (31, 33).

As described above, in oncogene-induced senescence (OIS) cells (fibroblast cell line IMR90), DOT1L was shown to inhibit the senescence-associated secretory phenotype (SASP) through epigenetic regulation of IL1A while still retaining the desired inhibition of proliferation. Therefore, inhibiting of DOTL may be an efficient way of inhibiting the negative side effects of senescence (74).

**Figure 1:**
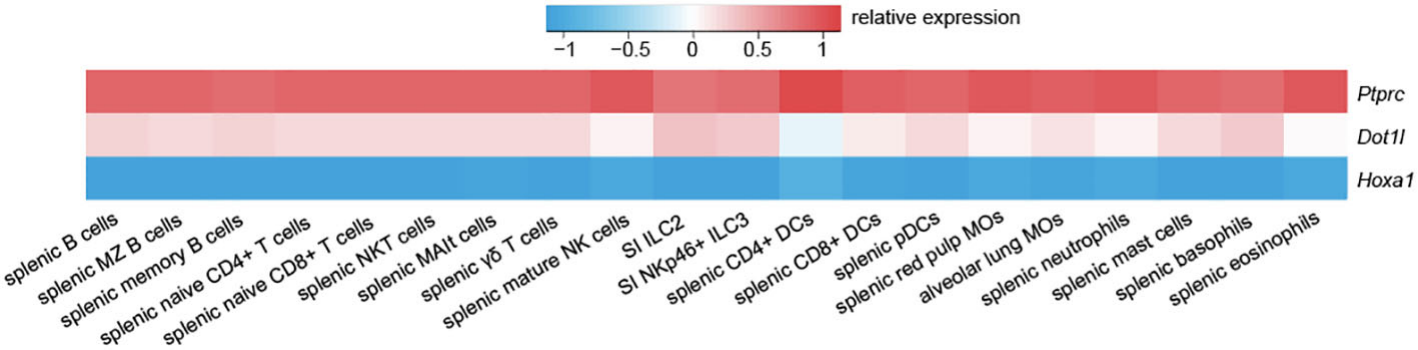
*Dot1l* exhibits a consistent expression pattern across the murine immune system. Compared to lowly expressed *Hoxa1* and highly expressed *Ptprc* (CD45), *Dot1l* shows a moderate and consistent expression pattern across the immune system. Data is adapted from GSE37448 (96).

While DOT1L expression is comparable across all immune cell subsets, in the innate immune system, DOT1L is highest expressed in basophils, mast cells, and in small intestinal type 2 innate lymphoid cells (ILC2) (**Figure 1**). However, its role in these subsets has not yet been defined. In particular, its role in fat mast cells, where DOT1L is highest expressed in the immune system, could shed a new light on epigenetic regulations in the pathophysiology of obesity. This is especially interesting on the basis that DOT1L limits inflammation and the fact that proinflammatory cytokines may increase the risk for obesity. Interestingly, DOT1L is a regulator of thermogenic adipocyte differentiation and function, and is thereby a key epigenetic modifier in obesity (97), showing that DOT1L may pose a highly interesting target (e.g. by stabilising its activity or by providing sufficient substrate) in obesity. It will therefore be of great interest to understand how inhibition of DOT1L affects the onset and maintenance of inflammatory processes during obesity.

Natural killer (NK) cells are innate immune cells that play a key role in the elimination of transformed cells and during tumour inflammation (98, 99). Functionally, NK cells are potent producers of IFN-γ and release an array of cytolytic molecules when activated, which play key roles in tumour control. NK cells are classified as ILCs and form a subgroup together with type 1 ILCs (ILC1), each with their own distinct functions and developmental pathway (100). Due to their presence in the TME and their significant role in killing metastatic cells, the role of epigenetic modifiers in NK cells has now been an area of intense research efforts (101). However, despite the use of DOT1L inhibitors in the clinic for patients with leukaemia, its role in NK cells and ILC1s has not been defined yet. We therefore investigated the role of DOT1L in NK cells and identified DOT1L as a gatekeeper of NK cell phenotype and function (37). Our results show that NK cells acquire an intILC1 phenotype independently of TGF-β [contrary to (102)] and show upregulation of markers found in ILC2 and ILC3, thereby providing additional evidence for its possible role in maintaining lineage integrity. However, and despite being highly expressed in ILC2 and ILC3 (**Figure 1**), the role of DOT1L in these subsets is pending in-depth investigations.

## Adaptive immune system

### B-lymphocyte development & V(D)J recombination

To date, there have been very few studies investigating the role of DOT1L in the context of lymphocyte development, however we have acquired a constellation of evidence over recent years that points to an important role for DOT1L during both B-cell and T-cell lymphopoiesis.

In 2010, *Xu* and colleagues elegantly demonstrated that H3K79me2, along with several other key post-translational modifications (PTMs), is dynamically modulated around key genes marked for V_L_-J_L_ recombination and that the addition of these modifications is dependent on pre-BCR signalling (44). The authors hypothesised that germline transcription of the genes which encode the immunoglobulin light chain, prior to their recombination, enables the recruitment of important histone modifiers which bind to the transcriptional machinery during initiation or elongation to mark relevant sites for the recombination event (44). If this theory holds true, it remains to be determined how H3K79me2 contributes to the recombination process and whether, like H3K4me3, lysine 79 methylation enables the docking of the RAG recombinase machinery through direct recognition, whether the mark is simply present to assist with the eventual transcription of the recombined construct or whether it might be participating via an alternative process entirely (103, 104). Moreover, this work is constrained by the limitations involved with studying H3K79me2 at single cell resolution via modern ChIP-sequencing methods, such as single cell CUT&Tag, that have now been optimised for other histone marks (105). Bulk ChIP-sequencing approaches are unable to answer specific questions involved with how each cell selects its unique combination of V(D)J genes and whether the use of histone modifiers and their modifications is uniform across all developing B-cells or variable depending on the specific cell or the recombined gene segment. It will also be important to perform other analytic approaches in parallel, such as ATAC-seq, Hi-C and ChIP of other marks and transcription factors, to gain a mechanistic understanding of how H3K79me2 is facilitating and contributing to the recombination process.

Work published in 2020 by *Aslam et al.* and *Kealy et al.* provided further evidence for a key role for DOT1L during B cell development by employing a *Dot1l* ^f/f^ *Mb1* ^Cre/+^ mouse model to delete *Dot1l* at the pro B cell stage of development (42, 43). This conditional knockout resulted in a severe decline in pre B-cell numbers and a resulting reduction in all subsequent subsets leading to a diminished mature B-cell pool. Those *Dot1l*-deficient B-cells that did make it to maturity were still capable of generating low titres of antibodies, assembling and organising themselves correctly within the secondary lymphoid organs and generating antigen-specific immune responses to immunisation and infection (42, 43). What separates the B cells that require DOT1L for their formation from those that can reach functional maturity in its absence requires further elucidation along with the precise mechanism by which DOT1L is achieving its role during early B cell lymphopoiesis.

### Humoral immune response

When mature B cells become activated and commence a humoral immune response, they begin by dividing and changing their gene expression program before branching off into an array of distinct developmental pathways. Together, the resulting B cell progeny are able to deliver a well-rounded and potent humoral response comprised of rapidly-responding, albeit short-lived, antibody secreting cells as well as higher-quality, longer-lived descendants that form later in the response via the Germinal Centre (GC) reaction. GCs are highly organised, dynamic structures wherein responding B cells undergo cycles of proliferation, somatic hypermutation and affinity-based selection to stochastically and scholastically transform into high-affinity clones. If selected, these clones will have the capacity to become highly-neutralising plasma cells and memory B cells with the potential to provide lifelong protection to their host. These events require stringent instruction by a team of epigenetic regulators and transcription factors working together to precisely modulate the events of clonal expansion, localised somatic hypermutation and differentiation as swiftly as possible whilst attempting to minimise the risk of cancer that is implicitly heightened by these biological processes (106, 107).

Research into the role of DOT1L in regulating the humoral immune response was pioneered by *Aslam et al.* and *Kealy et al.* using an *Mb1* ^Cre/+^ and a *Cd23* ^Cre/+^ mouse model to induce a conditional *Dot1l* deletion in, respectively, the Pro and transitional B cell subsets, prior to their becoming mature naive B cells (35, 42). These mice were subsequently challenged with a range of immunogens as well as several models of a *bone fide* infection, such as influenza and the Lymphocytic Choriomeningitis virus (LCMV), to assess the effects of deleting *Dot1l* on antigen-dependent B cell differentiation in different contexts. A consistent and striking feature of the *Dot1l-*deficient T cell-dependent humoral immune response was a total absence of GCs (42, 43). Further analysis revealed that B cells with a deletion of *Dot1l* were still capable of B cell activation, migration to the T:B border and could even express the master transcription factor (TF) of the GC program, BCL6, at normal levels, however failed to localise back into the B cell follicles for GC assembly (42). Whilst informative, these experiments were unable to answer if and how DOT1L might be further contributing to the maintenance and survival of the fully-formed GC. *Duan et al.*, however, were able to shed some light on this by using the Burkitt’s Lymphoma-derived Ramos cell line to show that DOT1L appears to cooperate with AID to achieve one of the central tenets of the GC: somatic hypermutation of the immunoglobulin genes (45). The precise contribution of H3K79me2 to AID-mediated somatic hypermutation and whether these observations are conserved in the context of an *in vivo* GC reaction has yet to be established. From these studies, it is now clear that DOT1L has joined the ranks of numerous other documented epigenetic regulators of the GC reaction. However, further work is needed to determine how these different key players cooperate to achieve the unique conditions necessary for affinity maturation.

In contrast to the indispensable role for DOT1L in the context of the GC reaction, plasma cell differentiation, survival and function are not dependent on the presence of DOT1L either *in vitro* or *in vivo* (42, 43). There is a considerable reduction of *in vivo* plasma cells and a depletion of class-switched antibody-secreting cells responding to T-cell-dependent antigens in the absence of *Dot1l*, however this is hypothesised to be due to the absence of GC-derived plasma cells. IgM titres within the serum and the numbers of plasma cells that secrete this unswitched antibody isotype in response to NP-KLH immunisation were entirely unaffected by the loss of *Dot1l in vivo* (42). When mice were immunised with a T-independent antigen, NP-Ficoll, reduced antigen specific plasma cells and serum antibodies were observed *in vivo*, both unswitched and IgG3-switched, however this decrease was hypothesised to be due to a matching preliminary reduction in marginal zone B-cell pool in the *Dot1l*-deficient mice rather than a consequence of any role for DOT1L on plasma cells (42, 43). When naive B cells were removed from the mice and stimulated *ex vivo* with CD40L, IL-4 and IL-5, plasma cell differentiation was amplified in the *Dot1l*-deficient B cells (42, 43). It remains unclear why this increase was observed *ex vivo* and exclusively in this context. However, one may speculate that when the *in vivo* environment necessary to support the numerous branches of B cell fate is removed and *Dot1l* is deleted, B cells shed their epigenetic multilineage potential which in turn takes the brakes off direct plasma cell fate commitment. These findings raise interesting questions regarding the context-specific roles for histone modifiers like DOT1L in driving immune cell fate decisions during both effective and dysfunctional immune responses.

*Milcarek et al.* demonstrated that plasma cells produced in culture do possess greater H3K79me2 activity around enhancers surrounding *IgH*. However, any involvement of DOT1L in the regulation of antibody secretion appears to be functionally dispensable as shown by the conditional knockout studies described above (35, 42, 108). The expendability of DOT1L for plasma cell formation or function is also reflected by the drop in *Dot1l* mRNA expression as GC B cells transition into plasma cells, as highlighted by the ImmGen dataset (96). However, further work is required to elucidate the extent to which DOT1L might be contributing to plasma cell fate commitment by restraining entry into this subset or if it is indeed playing a redundant role at the terminal, antibody-secreting stage of B cell differentiation.

### CD4^+^ T cells

The deletion of DOT1L early in T cell development (LckCre-DOT1L^fl/fl^) leads to an almost complete absence of CD4^+^ but not CD8^+^ T cells, highlighting the importance of DOT1L for CD4^+^ T cell development (35). We have previously shown that DOT1L is a key regulator of CD4^+^ T helper (Th) cell development, function and lineage integrity by using CD4Cre-DOT1L^fl/fl^ mice, which delete DOT1L at a later developmental stage than by using LckCre-DOT1L^fl/fl^ mice (31, 33). Here, lack of T cell-intrinsic DOT1L resulted in lymphopenia, increased activation and disruption of CD4^+^ T cell development, function and lineage integrity (33). The deletion or inhibition of DOT1L methyltransferase activity in murine CD4^+^ T cells increased Th1 cell differentiation and IFN-γ production *in vitro*, suggesting that DOT1L is a negative regulator of the Th1 cell transcriptional program (33). In the absence of DOT1L in CD4^+^ T cells, TFs involved in repressing the Th1 cell differentiation program, such as BACH2, FOXP3 and NR4A3, were downregulated (31, 33, 109–113). The increased bias towards Th1 cell differentiation in DOT1L-deficient CD4^+^ T cells may be due to the loss of H3K79me2 coverage at these genes, limiting their expression and, therefore, ability to repress Th1-associated genes such as *Ifng* (33). These two publications (33, 35) clearly demonstrate the importance of DOT1L in CD4^+^ T cell commitment early in development but also shows that, as soon as CD4^+^ T cell are committed to their lineage, DOT1L is largely dispensable for CD4^+^ T cell survival but is responsible for the maintenance of transcriptional integrity.

Another possible reason for the phenotype in DOT1L-deficient CD4^+^ Th cells was the interaction of DOT1L and the PRC2 complex (31, 114–116), as hypothesised in B cells (43). T cell-specific deletion of EZH2, a member of the canonical PRC2 complex, shows a similar phenotype of increased IFN-γ production under Th1 polarising conditions (114). However, this study did not observe a direct impact on the activity of the PRC2 complex in the absence of DOT1L H3K79 methylation, as there was no change in the global levels of H3K27me3 (33). Whether a change in H3K27me3 levels occurs at specific genes rather than globally is yet to be confirmed. A recent study showed that deprivation of methionine, such as in the TME, reduced DOT1L-dependent H3K79me2 in CD4^+^ T cells, leading to increased expression of the exhaustion marker PD-1 (32). This increased PD-1 expression was found to be due to DOT1L-H3K79me-dependent loss of AMPK expression. Ultimately this study highlights an epigenetic link between methionine metabolism and AMPK, with CD4^+^ T cell exhaustion in the TME. It is unknown however, whether inhibition or deletion of DOT1L in CD4^+^ T cells leads to increased Th1 differentiation and therefore IFN-γ production within the TME. This would be an important avenue to investigate for future studies given the Th1 biased phenotype observed in DOT1L deficient CD4^+^ T cells *in vitro*.

In addition to conventional CD4^+^ T cells, a recent study has identified DOT1L derived H3K79me to be involved in the thymic development of regulatory T (Treg) cells (117). A lack of the fatty acid, oleic acid, within the thymus resulted in the deposition of H3K79me2 at *Atp2a2* (encoding sarco-endoplasmic reticulum Ca2^+^-ATPase, important in controlling TCR signal transduction) and thereby promoting its expression in double-negative (DN)3 thymocytes. This led to an increase in Treg cell differentiation. The presence of the fatty acid, oleic acid, was shown to inhibit the enzymatic activity of DOT1L (117). This study highlights a link between fatty acids and epigenetic regulation during immune cell development and demonstrates DOT1L as a central player in this. Aside from this study, little is known about the role of DOT1L in Treg cell development, lineage integrity and function, therefore warranting further investigation. It would be interesting to further delve into DOT1L’s role in thymic Treg cell development, and whether it is also required to maintain the transcriptional profile of a Treg cell following differentiation. If this was the case, DOT1L could present as an alternative epigenetic target for destabilising Treg cells in the TME, where they act as a barrier to effective anti-tumour immunity. The generation of mice possessing a Treg cell-intrinsic deletion of DOT1L will be worthwhile for future studies to elucidate DOT1L’s mode of action in Treg cell biology.

### CD8^+^ T cells

DOT1L activity is not limited to CD4^+^ T cells but is also involved in many aspects of CD8^+^ T cell biology. DOT1L deletion during thymocyte development increased the frequency of CD8^+^ T cells with a virtual memory (TVM) phenotype (CD44^+^ CD62L^+^) seemingly at the expense of antigen naive CD8^+^ T cells (CD44^-^ CD62L^+^) (35). In this study *Kwesi-Maliepaard et al.* also demonstrated that in the absence of DOT1L mice failed to generate E7-specific CD8^+^ T cells following HPV-E7 vaccination. As described earlier, the authors identified a mechanism whereby the loss of DOT1L impacts EZH2 mediated H3K27me3 gene repression, a mechanism known to regulate CD8^+^ T cell differentiation (35, 118). Evidence that DOT1L also regulates TCR activation comes from a study by *Kagoya et al.* where the authors used the small molecule inhibitor SGC0946 to decrease T cell reactivity. The authors used this to alleviate allogeneic graft versus host disease (GVHD) responses *ex vivo* without hindering physiological T cell responses (36). Mechanistically, inhibition of DOT1L reduces miR181a expression leading to upregulation of DUSP6, a phosphatase known to inhibit T cell signalling (119). However, whether DOT1L plays a role in antigen-specific CD8^+^ T cells during viral infection remains unclear and requires further investigation.

The role of DOT1L in CD8^+^ T cells is not restricted to T cell differentiation/activation but it is also involved in homeostatic signalling pathways. *Bian et al.* demonstrated that the TME restricts methionine levels via the upregulation of the methionine transporter SLC43A2 (34). Decreased methionine levels severely impact DOT1L-mediated H3K79me in tumour infiltrating lymphocytes (TILs), leading to decreased STAT5 expression. Impaired STAT5 signalling resulted in impaired anti-tumour responses in the murine colon carcinoma 38 (MC38) tumour model via increased apoptosis of TILs (34).

Outside of these seminal papers, little is known about the function and role of DOT1L in CD8^+^ T cells. Some information can be inferred, for instance, analysis of publicly available RNA-seq datasets shows an increase in *Dot1l* transcript levels following T cell activation (120), suggesting that DOT1L may be involved in regulating CD8^+^ T cell activation. Considering its established role in CD4^+^ T cells and what is currently known in CD8^+^, inhibiting DOT1L may provide a therapeutic target for dampening adverse CD8^+^ T cell responses including those in autoimmunity whilst augmenting DOT1L function may facilitate heightened CD8^+^ T cell responses important in combating viral infections and cancers.

## Potential mechanisms

**Figure 2:**
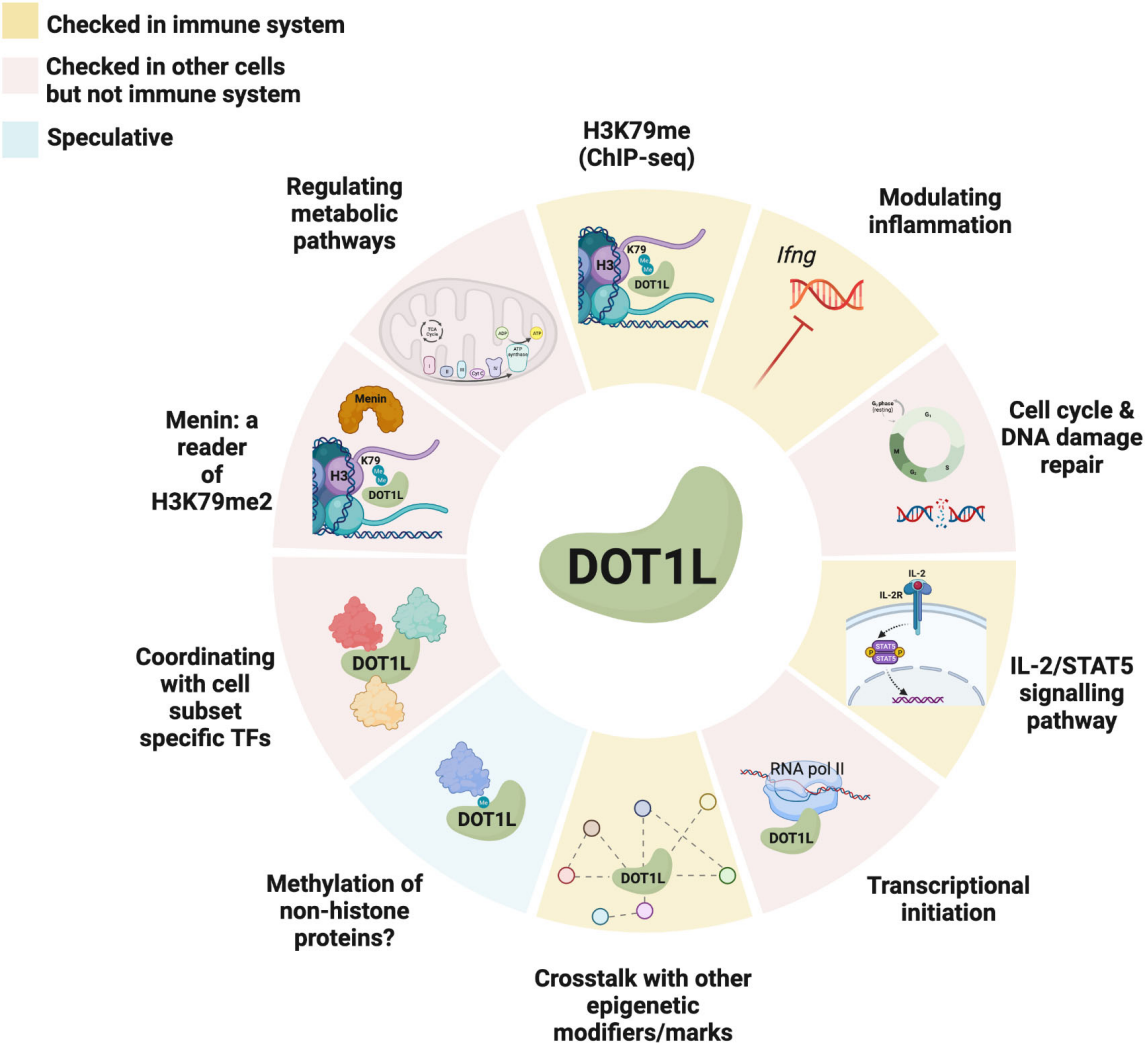
Known and potential mechanisms of action of DOT1L in immune and non-immune cells. Different tile colours represent studies that were performed in either the immune system (yellow), other cells (red), or are speculative (blue). Created with BioRender.com.

Epigenetics is still a relatively nascent field and while we have begun to gain some insight into the behaviour, context-specific functions, and methylation patterns of histone modifiers such as DOT1L, we have still yet to answer many of the fundamental questions revolving around the regulation of their conditional activity or of the precise consequences of H3K79 methylation. Hence, the definitive elucidation of DOT1L’s central purpose and distinctive contribution to the immune system, as well as various other biological contexts, remains an ongoing pursuit (**Figure 2**).

It remains controversial as to how DOT1L is recruited to distinct gene targets in different cell types operating under homeostatic conditions. Several studies have provided evidence of TF recruitment or involvement in driving the targeting of the H3K79 lysine methyltransferase (121), meanwhile the strong positive correlation between DOT1L and RNA polymerase II binding as well as the established interaction between DOT1L and the phosphorylated C-terminus of actively transcribing RNA polymerase II raises the possibility that the transcriptional machinery may be solely responsible for DOT1L’s recruitment and binding (122).

Additional unknowns include: if H3K79me2 or its reader, Menin, is providing a docking site for other key regulators in biological contexts such as V(D)J recombination or GC formation to enable their function? Moreover, if DOT1L is indeed cooperating with different TFs in different biological contexts, such as AID and Zc3h10, how are these context-specific collaborations mediated (45, 121)?

In 2002, *Ng et al.* published their ground-breaking work linking Dot1 to H3K79 methylation in yeast (58). One of the most intriguing aspects of their findings, as noted by the researchers at the time, is that they extend on the prevailing histone code hypothesis to accommodate the idea that regulatory cofactors can also read PTMs located within the globular region of histones and not just those found on the tail. More than 20 years later, we still do not have a concrete understanding of what unique biological advantage is bestowed by a histone mark that is located within the H3 globular region nor of which regulatory cofactors might be responding to it. As described above, novel technological advancements and approaches in recent years have begun to provide insight into the unique and conditional roles of H3K79 methylation and other histone marks in important biological contexts such as the immune system. However, how each of the numerous histone modifications specifically and mechanistically contribute to the epigenetic landscape remains to be determined. One feature that sets the suite of available histone marks apart is that they vary in their half-life and mitotic heritability; theoretically providing cells with a toolbox of marks with which to bookmark and manipulate chromatin at specific loci for variable amounts of time and at different stages of the cell-cycle. H3K79me2 is a comparatively persistent and heritable mark compared to H3 acetylation or phosphorylation (123, 124). Interestingly, *De Vos* and colleagues discovered that over the course of multiple cell divisions, H3K79 methylation state can be used as a ‘molecular clock’ to gauge histone age (125). This can be achieved since, when DNA is replicated and cells divide, some of the histones are inherited from the parent cell whilst others have to be generated *de novo.* Inherited histones often already carry PTMs such as H3K79me due to prior exposure to DOT1L in the parent cell. Notably, these inherited histones which already possess H3K79me will acquire additional layers of H3K79 methylation once re-exposed to DOT1L in the daughter cell while, at the same time, newly-formed histones at the same gene loci are being exposed to DOT1L for the first time and hence acquire their first H3K79 methyl mark. This results in a mosaic of different H3K79 methylation states at the same loci within daughter cells, unlike their parent cells, with an enrichment of H3K79me3 in the inherited H3 histones compared to newly generated histones that possess comparatively more H3K79me1 (125). The daughter cells used in the context of this study were not differentiating but simply clonally expanding, which assumes that the epigenetic state should be reconstructed identically after each mitotic division. Therefore, this leads us to conclude that the functional contribution of these differences in methylation states between inherited versus *de novo* histones are either negligible or undetectable by the assays performed thus far. To justify these differences between parent and daughter cells, some have theorised that marks like H3K79me2 may be read as a cluster rather than per nucleosome and, as a result, the slight differences in methylation states between neighbouring nucleosomes may be negligible to the reader (125, 126). However, this hypothesis requires further validation. It may even be the case that features such as this have been conserved for a specific biological reason such as offering cells an alternative way to track their own division history or to assist with nucleosome turnover. Of note, different mouse models have confirmed the conditional deletion of DOT1L by either showing a direct loss using western blot for the DOT1L protein or indirectly – as DOT1L is the only KMT for H3K79 - by showing the absence of H3K79me by either western blot or flow cytometry (31, 35, 42). This highlights that the assessed cells underwent multiple rounds of division and histone turnover to lose H3K79me.

Histone marks can contribute to gene expression via an array of different mechanisms. H3K79me2 has long been believed to be a key regulator of chromatin accessibility. However, more recent work via ATAC-seq has demonstrated that deleting DOT1L does not directly affect chromatin accessibility at promoter sites (70). Despite these findings, other studies have provided evidence of a clear effect on chromatin accessibility following the use of DOT1L inhibitors (53). Until we map the interdependency of the histone modifier network via cross-talk or the manipulation of one another’s gene expression, the indirect effects of DOT1L on chromatin accessibility in different biological contexts will remain elusive.

Quite consistently, when RNA-seq and H3K79me2 ChIP-seq are performed in parallel, the deletion or inhibition of DOT1L, in both homeostatic and malignant contexts, results in a decrease in mRNA expression of many of the genes that possess H3K79me2 in that cell type. However, this is not the case for all genes marked by DOT1L. There is varying functional redundancy for H3K79 methylation on certain genes in specific biological contexts. However, we are yet to understand how this is being mediated or its significance to those genes that would ordinarily carry H3K79 methylation yet remain functionally unaffected by – and possibly even protected from – the loss of DOT1L. Recent work has highlighted partial functional redundancy between DOT1L and the super elongation complex (SEC). By using a catalytically inactive form of DOT1L and thereby disrupting of DOT1L’s methylation potential, RNA Polymerase II binding was largely preserved. However, this is lost when the SEC was simultaneously blocked (69). This kind of layered overlapping of functional roles by different epigenetic regulators may be the explanation for why some genes and even some cell fates are preserved in the absence of DOT1L, while others are entirely reliant on DOT1L’s expression and function.

If we may venture further into the realm of speculation, one of the advantages of epigenetics is that it might help to explain how an adaptive immune memory cell’s prior experience shapes and improves its ability to respond to future encounters. H3K79me2 abundance is correlated with the transcriptional rate of elongation (127). In this way, DOT1L may be priming specific genes for faster recall in the context of immune memory responses. Epigenetic regulation of the histone code is likely to hold the key to revealing the enigmatic ability of memory cells to respond to restimulation more rapidly and with greater vigour than their naive counterparts, although entirely new technical approaches will need to be formulated to address these hypotheses.

DOT1L is playing critical roles across the immune cell network and its dispensability varies between contexts. Just as certain types of leukaemia hijack DOT1L to open up novel gene expression programs and pathways, we speculate that epigenetic regulators like DOT1L may have been similarly used to drive the evolution of novel immune differentiation pathways. In this way, modifiers such as DOT1L may indeed have pioneered some of the alternative immune cell fates that were later consolidated by the establishment of key TFs and which have gone on to underpin the heterogeneity and adaptability of the higher order immune system.

## Current problems and future directions

The identification of DOT1L’s significance and impact in the immune system is currently being investigated by many research groups around the globe. Research on the role of DOT1L in various cell types is of utmost importance as 1) the extrapolation of its significant impacts on various immune cell subsets, *e.g.* CD4^+^ T cells (33), 2) the realisation that the attempt to inhibit DOT1L in cancer cells with systemically acting small molecule inhibitors (SMIs) also has a profound effect on other cells (128), and 3) the scientific challenges that coincide with research on epigenetic modifiers, in particular DOT1L. There are currently significant technical limitations barring direct study of the role and mechanism of action of DOT1L since suitable reagents are sparse. For instance, there is only one commercially available antibody available that would allow for the detection of DOT1L in ChIP assays (38). As this antibody must be validated by other research groups, it is difficult to assess the location and potential interaction partners at the site of action without a reliable antibody against DOT1L. However, we know from studies on cancer cells that DOT1L has many interaction partners (87). Yet, a systematic study of the interaction partners of DOT1L in immune cells is missing, which would provide a chance to understand the non-enzymatic interactions of DOT1L. As there have not been studies on the subcellular localisation of DOT1L in immune cells under different conditions, it would be important to assess its potential role in different cell compartments. However, DOT1L is obviously nuclear due to its methyltransferase function and due to its nuclear interaction partners, as assessed by mass spectrometry and co-immunoprecipitation experiments (83, 87). These studies would then provide the possibility to allow for the identification of specific interaction partners for each immune cell subset and their potential targeting to improve cell-specific function. In addition, the availability of suitable antibodies would also allow for the spatiotemporal role of DOT1L in gene regulation, specifying its potential role as transcription elongator or initiator of gene transcription. While there are reliable antibodies against H3K79me2 to assess the presence of this epigenetic mark in the genome using ChIP assays, few attempts have been made to assess the presence of H3K79me2 using flow cytometry on a global level (39, 43). Analysing the coverage of H3K79me in ChIP-assays or by flow cytometry is particularly challenging due to the mandatory use of SDS, which is needed to unfold the core of H3 to make it accessible for antibodies. This technical challenge has prevented us thus far from analysing H3K79me at a single-cell level.

Small molecule inhibitors are an elegant way to study the methyltransferase activity of methyltransferases, such as DOT1L, in the immune system (31). However, despite their use in the clinical setting (29, 129), these molecules inhibit the methyltransferase activity not only in the intended cells, such as cancer cells, but - due to their membrane-crossing capacity - inhibition is also observed in bystander cells. Therefore, SMIs are not the ideal drug for the selective inhibition of DOT1L, despite moderate yet temporary effects (29). Ideally and mainly to avoid unwanted side effects in other cells or tissues, these inhibitors would be delivered by a targeted approach for precision medicine, such as with antibodies or in nanoparticles (130). These targeted deliveries would possibly allow for great success in precision medicine, *e.g.* the targeting of DOT1L in Th2 cells for the treatment of allergic reactions, as suggested earlier (33). Another possibility would be the targeting of DOT1L for degradation, or impairing the binding of DOT1L to its targets, but exact mechanisms of action for these types of interventions remain the focus of intense study. Considering the adaptability and breadth of functions demonstrated by DOT1L in a range of different cell types, both histone methyltransferase-dependent and independent, it is likely that targeting the stability of DOT1L will be a desirable therapeutic strategy for an array of different pathologies. Here, targeting the interaction of DOT1L with SIRT1 (87) could prove highly interesting in the field of ageing: DOT1L could degrade SIRT during autophagy, act as a guide to its location, not have an effect on the SIRT1 network, or may even increase stability of SIRT1. Answers to these questions could result in interesting new findings about cell ageing.

According to public datasets (**Figure 1**), *Dot1l* is highest expressed in mast cells and basophils across the immune system (96). However, there is currently no publicly available data that directly focuses on the significance and impact of DOT1L in mast cells or basophils. Crossing of DOT1L floxed mice with specific Cre mice such as Mcpt5/Cma1-DTR (131) (mast cells) or Mcpt8-Cre (132) (basophils) would be a relatively easy way to address DOT1L’s role in these cells. This could be especially interesting since these cells are pivotal to fat mast cells as described earlier.

## Conclusion

Initially identified and studied in yeast as a disruptor of telomeric silencing (4, 5), DOT1L has made its scientific journey to become one of the most promising therapeutic targets to combat unfavourable gene expression in patients with MLL*r* leukaemia and adult acute leukaemia (AAL) (29, 129) and is now under intense investigation for its critical importance in orchestrating the immune network along with other biological processes that fall outside the scope of this review. Overall, DOT1L seems to regulate the maturation and activation of immune cells in a broad array of contexts as well as repress proinflammatory processes. It is difficult to pin down an overarching role for this versatile and perplexing KTM, however it is clear that from the innate to the adaptive branches of the immune system, DOT1L is consistently playing a central role in making important fate decisions and orchestrating changes to the transcriptional program. Future directions of research around DOT1L in the immune system are manifold, ranging from further elucidation of its role across the immune cell network to more specific questions surrounding the molecular mechanisms of gene regulation and finally to exploration of the potential to restore immunological imbalances via targeting DOT1L. Overcoming the limitations precluding full characterisation of the role and functional consequences of key immune regulators like DOT1L are essential to make great medical strides towards the development of accurate and informative *in silico* models and strategies to remodel the epigenetic landscape for the treatment of immune cell dysfunction.

## Author contributions

SS: Conceptualization, Data curation, Formal analysis, Funding acquisition, Investigation, Methodology, Project administration, Resources, Supervision, Validation, Visualization, Writing – original draft, Writing – review & editing. LK: Conceptualization, Investigation, Resources, Validation, Writing – original draft, Writing – review & editing. JR: Conceptualization, Investigation, Resources, Validation, Visualization, Writing – original draft, Writing – review & editing. DT: Conceptualization, Investigation, Resources, Validation, Visualization, Writing – original draft, Writing – review & editing.
